# A Novel, Precise and High-Throughput Technology for Viroid Detection in Cannabis (MFDetect^TM^)

**DOI:** 10.3390/v15071487

**Published:** 2023-06-30

**Authors:** Angel Fernandez i Marti, Marcus Parungao, Jonathan Hollin, Berin Selimotic, Graham Farrar, Tristan Seyler, Ajith Anand, Riaz Ahmad

**Affiliations:** 1 Department of Environmental Science, Policy and Management, University of California, Berkeley, CA 94720, USA; 2 MyFloraDNA, Inc., 1451 River Park Dr., Sacramento, CA 95815, USA; marcus@myfloradna.com (M.P.); jonathan.hollin@myfloradna.com (J.H.); bselimotic@myfloradna.com (B.S.); ajith.anand@myfloradna.com (A.A.); riaz@myfloradna.com (R.A.); 3 Glass House Farms, 645 W Laguna Road, Camarillo, CA 93012, USA; graham@glasshousegroup.com (G.F.); tristan@glasshousegroup.com (T.S.)

**Keywords:** hop latent viroid, cannabis, viroids, RT-LAMP, RT-qPCR, MFDetect^TM^, cannabis pathogen

## Abstract

Hop latent viroid (HLVd) is a severe disease of cannabis, causing substantial economic losses in plant yield and crop value for growers worldwide. The best way to control the disease is early detection to limit the spread of the viroid in grow facilities. This study describes MFDetect^TM^ as a rapid, highly sensitive, and high-throughput tool for detecting HLVd in the early stages of plant development. Furthermore, in the largest research study conducted so far for HLVd detection in cannabis, we compared MFDetect^TM^ with quantitative RT-PCR in a time course experiment using different plant tissues, leaves, petioles, and roots at different plant developmental stages to demonstrate both technologies are comparable. Our study found leaf tissue is a suitable plant material for HLVd detection, with the viroid titer increasing in the infected leaf tissue with the age of plants. The study showed that other tissue types, including petiole and roots, were equally sensitive to detection via MFDetect^TM^. The assay developed in this research allows the screening of thousands of plants in a week. The assay can be scaled easily to provide growers with a quick turnaround and a cost-effective diagnostic tool for screening many plants and tissue types at different stages of development.

## 1. Introduction

*Cannabis sativa* L. is a valuable plant which has been widely cultivated for its industrial [1] ornamental [2], nutritional [3], medicinal, and recreational [4] potentials. From regulatory and application perspectives, cannabis plants are categorized based on the level of Δ9-tetrahydrocannabinol (THC), one of the most important phytocannabinoids [5]. Plants are generally classified and regulated as industrial hemp if they contain less than 0.3% THC in the dried flower (this level varies by country) or specific drug-type with more than this threshold [6].

Hop latent viroid (HLVd) is an emerging disease in cannabis that significantly affects plant vigor, yield, and bud quality. The viroid was first found and isolated from two commercially grown hop cultivars in North Spain [7]. It has been reported that although HLVd-infected hop plants are asymptomatic, it can cause substantial yield loss and decrease both bitter acid contents and terpenes [8] HLVd has also been found in stinging nettle *(Urtica dioica*) and two cultivars of hop, including commercial hop (*Humulus lupulus*) and Japanese hop (*Humulus japonicus* Sieb. and Zucc.). The viroid was initially reported in cannabis in 2019 in California from two independent studies [9,10]. A 2021 survey from Dark Heart Nursery Research concluded that 90% of cannabis facilities in California were infected with HLVd. Their recent report states that HLVd is a hidden threat and most likely the biggest concern for cannabis and hop growers worldwide (https://mjbizdaily.com/experts-sound-alarm-over-global-spread-of-cannabis-viroid/ accessed on 26 May 2023). There are estimates that HLVd could result in crop losses of up to USD 4 billion annually for the cannabis industry [11].

Viroids are the smallest known protein-free infectious RNA molecules that predominantly cause plant disease [12]. They rely on host-cell DNA-dependent RNA polymerase and processing enzymes for replication and pathogenesis [13]. HLVd is a small, circular RNA molecule of approximately 256 nucleotides, including a conserved central region (CCR) and terminal conserved hairpin (TCH) [11,14]. The symptoms produced by the viroid depend upon the host plant species and viroid variant [14]. In cannabis, the viroid is associated with “dudding” or “duds” disease of cannabis, which is one of the most devastating cannabis diseases (sy. Hemp) (*Cannabis sativa*, *Cannabis indica* and *Cannabis ruderalis*). Once infected, the viroid spreads rapidly through cannabis plants, leading to stunted growth, weaker flower smell, diminished flower quality, lower yield, and up to 50% reduction in cannabinoid and terpene production [9,10,11,15].

HLVd is very stable in plants and is easily transmitted by vegetative propagation or mechanically through contaminated tools [16]. Due to the non-coding nature of viroids, they recruit host DNA-dependent RNA polymerase II for replication. The resulting replication is error-prone, creating “quasi-species” or sequence variants [15]. Comparative analysis of all HLVd sequences isolated from cannabis plants identified two distinct HLVd isolates, isolates Can1 (GenBank Acc. No.: MK876285) and Can2 (GenBank Acc. No.: MK876286) [10]. The Can1 isolate has 100% sequence similarity with HLVd species [17], while the Can2 isolate has a point mutation, U225A [10]. Interestingly, both isolates are infectious and have been reported from HLVd-infected cannabis plants in the USA [9,18].

The viroid can remain latent, and plants remain symptomless for an extended period. As a result, infected plants can be accidentally propagated, spreading the viroid and making it hard to eradicate from infected plants and growing areas. Early screening of plants and implementation of integrated pest management (IPM) seem to be the most efficient means of limiting the spread of the disease [16,19,20]. The early detection of infected plants and remediation is critical to reducing the rate of infectivity, the spread of the viroid, and minimizing losses.

Several technologies have been developed and used to detect viroids in plants. The methods include bioassay, nucleic acid hybridization [21], return-polyacrylamide gel electrophoresis [22], RT-qPCR [23,24,25], and reverse transcription loop-mediated isothermal amplification (RT-LAMP) [26,27,28,29]. Of these, RT-qPCR and RT-LAMP are considered to be standard procedures. Nevertheless, both have their advantages and drawbacks. RT-qPCR is a very accurate method for pathogen detection; however, this technique is expensive, requiring sophisticated equipment, expert personnel to perform the test, and it lacks high throughput capability. RT-LAMP is gaining a lot of popularity for pathogen detection in plants, animals, and humans. The advantage of RT-LAMP is its capability for high throughput at low cost, and turnaround times can be rapid [26]. It provides simple visual colorimetric detection techniques that bypass the need to use a sophisticated analytical instrument. RT-LAMP uses four to six primers to target and amplify a specific region of the pathogen, and the reaction occurs isothermally in a single tube. RT-LAMP can be designed for detecting multiple viroids using a generic primer or universal set of primers [30,31].

The virulent nature of this viroid in cannabis and its high rate of transmissibility and spread across most of the gardens in California demand a novel approach that combines the robust and accurate detection of RT-qPCR and the low-cost and high throughput of RT-LAMP to allow containment of viroid spread within California and to other states in the country and cultivation areas worldwide. Here, we describe such an approach that MyFloraDNA, Inc. has developed and named MFDetect^TM^. Furthermore, there is currently an open public debate about what is the best plant tissue for pathogen detection, so in this study we aim to resolve this by testing our approach and RT-qPCR on a range of plant tissues.

Therefore, the objectives of this study include: (1) developing a hybrid method combining the ease of RT-LAMP and sensitivity of RT-qPCR for highest accuracy, high-throughput, and low cost, (2) comparing the accuracy of MFDetect^TM^ with RT-qPCR for HLVd detection, (3) identifying a preferred tissue type for early viroid detection during development of the plants.

## 2. Material and Methods

### 2.1. Development of the MFDetect^TM^ Method

MFDetect™ is a proprietary technology developed by the MyFloraDNA research team for plant pathogen detection. This innovative testing platform combines high-throughput handling procedures, RT-LAMP, and quantitative fluorescence measurements utilizing a real-time PCR machine (Applied Biosystems ViiA 7, Waltham, MA, USA) to facilitate rapid, high volume, sensitive and specific detection of numerous pathogenic viruses, viroids, and fungi. For HLVd detection, we designed primers (Table 1) using the whole genome sequence for hop latent viroid retrieved from the NCBI database (EF613183.1). The primers were designed in-silico (Primer Express 3.0) to work at high annealing temperatures, resulting in highly specific target amplification and elimination of nonspecific targets. After addition of MFDetect™ proprietary integrating dye, end-point digital fluorescence measurement enables relative quantification of infection between positive and negative samples, offering an alternative measurement to Ct values associated with RT-qPCR analysis. Primers were synthesized by Integrated DNA Technology, prepared in nuclease-free water, and stored at −20 °C.

Initially, these primers were tested on synthetic DNA designed from the HLVd genome and subsequently HLVd-positive and HLVd-negative plant samples were confirmed visually by our nursery collaborators. After confirming that the primers only amplified the HLVd genome and there was no nonspecific amplification of other viruses, viroids, or cannabis genome, the primers were selected for testing HLVd in cannabis on a large scale.

For the MFDetect™ protocol, 80–100 mg of fresh leaf, petiole, and root tissues were collected in 2 mL Eppendorf tubes. Beads were added to each tube and the tissue was ground to powder in liquid nitrogen using a TissueLyser (Qiagen, Hilden, Germany) for 5 min. Five hundred microliters of RNA Extraction Buffer, developed by MyFloraDNA, was added immediately to preserve the RNA integrity. Extracted RNA was diluted further at a 1 to 50 ratio using nuclease-free water. From the diluted RNA, 5 µL were used for cDNA conversion and amplification of the HLVd genome in the 20 µL reaction volume using a commercial master mix and enzymes (ThermoFisher, San Jose, CA, USA), and proprietary primers.

Reactions were prepared in 96 well plates with two HLVd positive controls and two no-template controls (NTC) in each plate. After an initial amplification, PCR products were validated and quantified further by ViiA7 qPCR (Applied Biosystem) using the FAM channel for the reporter dye and Low Rox as a passive dye to normalize the reporter signal. Delta Rn values were calculated to quantify the magnitude of the fluorescence signal generated during the PCR at each time point. Delta Rn for MFDetect™ was calculated and compared in three plant tissues i.e., leaf, petiole, and root.

### 2.2. Validation of MFDetect^TM^ by RT-qPCR

For HLVd testing with TaqMan RT-qPCR (Norgen, Thorold, ON, Canada), we extracted RNA from leaf, petiole and root tissue using a RNeasy Plant Mini kit (Qiagen, Hilden, Germany), quantified RNA with a NanoDrop and used 15 ng of RNA in a total volume of 20 µL reaction mixture using the manufacturer’s protocol and ran through ViiA7 qPCR (Applied Biosystem). In each 96 well plate, we ran four HLVd positive and two HLVd negative samples as controls. VIC was used as a passive reference dye to normalize the reporting signal of the FAM dye. Ct values were recorded when the positive samples crossed the threshold level.

We collected leaf tissue from 5030 cannabis plants, including multiple cultivars, and at different plant growth stages (5–10 weeks old) from our partner Glass House Farms. After an initial screening of these 5030 plants, we selected forty-four HLVd-positive plants and six HLVd non-infected plants from the 5–6-week-old stage and tested them again using the classic RT-qPCR protocol.

### 2.3. Identifying a Preferred Tissue Type for Early Viroid Detection during Development of the Plants

We aimed to determine the variability of the HLVd load in three different tissue types: leaves, petioles, and root tissue. For that, we collected leaves, petioles and roots and placed them in separate tubes from each of the fifty plants making a total of 150 samples to be tested. In addition to testing different tissue types, we wanted to analyze the same plant material using two different technologies, TaqMan RT-qPCR (Norgen, Canada) and MFDetect^TM^, and for this we duplicated the number of samples to 300 (150 for RT-qPCR including roots, petioles, and leaves and 150 for MFDetect^TM^ including roots, petioles and leaves).

To understand the progression and virulence of the pathogen through the growth of the plant, we used the same parameters as in the previous experiment that included the three different plant tissues (leaf, petiole and root) but added a new hypothesis –how soon and at what concentration we can detect the pathogen. For this, the experimental design included three different ages of the plants (5–6 weeks old, 7–8 weeks old and 9–10 weeks old) making a total of three-hundred samples to be analyzed. Considering all the parameters and hypotheses, within this study, a total of 600 samples were analyzed, which represents the largest research study conducted so far for HLVd detection in cannabis.

## 3. Results

### 3.1. Development and Optimization of the MFDetect^TM^ Assay

**Figure 1:**
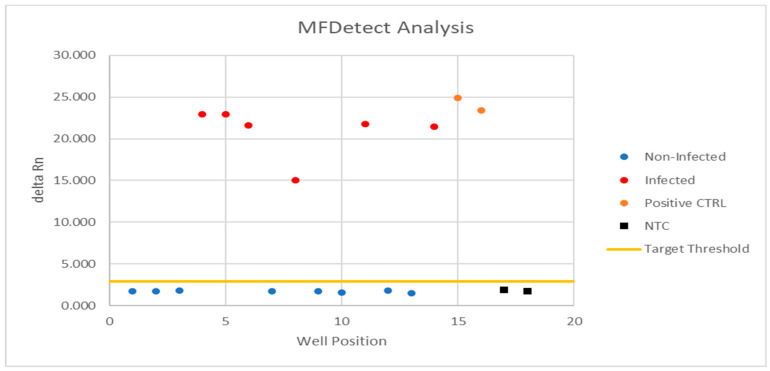
Plot representation of positive and negative detection using MFDetect^TM^.

MFDetect^TM^ primers amplified only in the HLVd positive controls as well as in the synthetic HLVd DNA, but not in the HLVd negative samples nor in the cannabis genome. We also optimized the amount of RNA for the RT-LAMP reaction by running a serial dilution experiment and selected the 40 times dilution as the best for the RT-LAMP reaction. We tested four different dyes to visualize and quantify the amplification products. We picked one that gave us confirmed positive and negative results that can be validated by RT-qPCR as seen in Figure 1.

### 3.2. Comparison of MFDetect^TM^ and RT-qPCR

**Figure 2:**
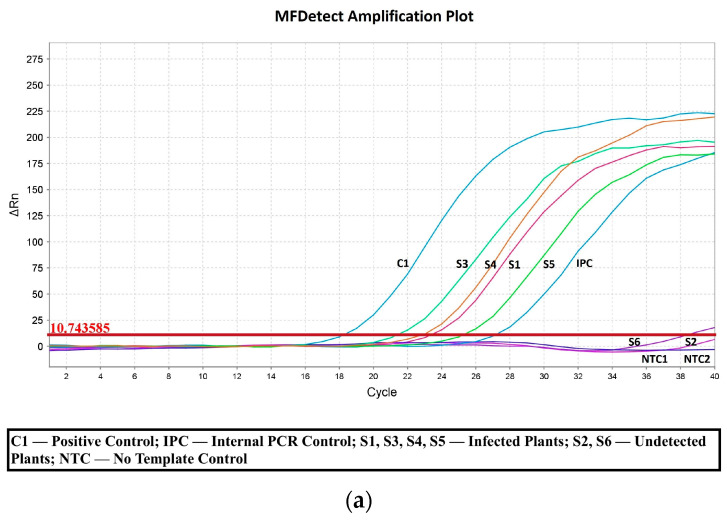

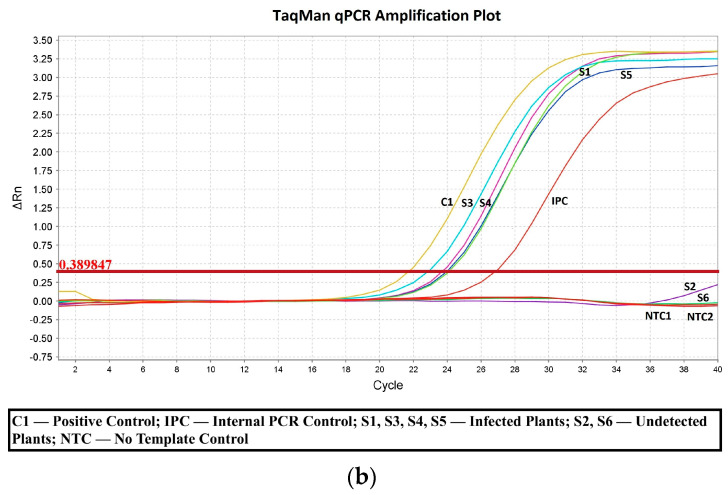
(**a**) Standard curve for MFDetectTM and (**b**) RT-qPCR using the leaf samples. Amplification plots showing the; (**a**) standard curve of the ΔRn values and (**b**) standard curve of the Ct values of the infected (S1, S2, S3, S4), uninfected (S5, S6), positive syn DNA control (C1), internal positive control (IPC), no-template control (NTC) used in the experiment.

To further validate MFDetect^TM^ sensitivity and accuracy in detecting HLVd, we conducted side-by-side experiments comparing MFDetect^TM^ and RT-qPCR techniques in forty-four infected and six non-infected plants. The ΔRn and Ct values from a selected number of plants were plotted to create the standard curves as shown in Figure 2. The standard curves for the different samples with MFDetect^TM^ and RT-qPCR were similar. As expected, the infected samples had higher ΔRn values (MFDetect^TM^) or lower Ct values (RT-qPCR), while the uninfected plant samples were below the detection limits. The data demonstrates MFDetect^TM^ is highly accurate and sensitive for detecting HLVd-infected plants. The ease of sample preparation, lower cost, and high throughput make MFDetect^TM^ a powerful HLVd detection technique for viroid presence in plants.

### 3.3. Identifying a Preferred Tissue Type for Early Viroid Detection during Plant Development

To determine the presence and variability of the viroid by tissue type and age of the plants, three different tissue types—leaf, petiole, and roots—were sampled biweekly from the forty-four infected and six uninfected plants. All the tissue types were collected in duplicate to compare side-by-side MFDetect^TM^ and RT-qPCR. A total of 600 samples were analyzed in this study and the data are summarized in Table 2.

With MFDetect^TM^ we noticed positive detection in 132/132 (100%) of the leaf samples, 86/88 (98%) of the petioles, and 84/88 (95%) in the root samples, respectively. While for the RT-qPCR assay, the positive detection ranged from 87/88 (99%) in leaves, 86/88 (98%) in petioles, and 88/88 (100%) in roots. The above results indicated that leaves are equally suitable for detecting HLVd from the infected plants compared to petioles and roots. Our results further strengthened the accuracy of MFDetect^TM^, achieving > 99% accuracy compared to RT-qPCR (Table 3).

The viroid load in the infected plants by tissue type and age was estimated by comparing the average ΔRn and Ct values, respectively. We noticed an apparent increase in the ΔRn values for the leaf material from 5–6-week-old material to the 9–10-week-old plants. The increase in ΔRn values ranged from 1.9× (7–8-week-old plants) to 3× (9–10-week-old plants) compared to values in the samples from the 5–6-weeks old plants. Thus, suggesting the viroid load in the leaf samples increased over time. A similar trend was observed with the Ct values in the duplicated leaf samples analyzed with RT-qPCR. A 2.2 average Ct value drop in 9–10-week-old plants was observed, compared to the Ct values in the 7–8-week-old plants. Which again, reinforces that the viroid load increased in the leaves over time. However, no such increase in viroid load was observed in the petiole and root tissues (Table 2). The above observation further suggests that the viroid replicates in the leaf tissue and is translocated into other parts as the plant ages. Our study indicates leaf is a good candidate and most likely the ideal tissue for HLVd detection with MFDetect^TM^ and RT-qPCR.

Concerning the analysis conducted to investigate the infection rate during plant development, our results obtained from one hundred fifty leaf samples (132 from infected and 18 from uninfected plants) indicates that the 132 infected samples matched 99.9% when compared over time (5–6, 7–8 and 9–10 weeks old). Whereas sixteen out of eighteen (89%) matched for the uninfected plants. Our data indicated that MFDetect^TM^ achieved 99.9% accuracy in detecting infected plants as early as week 5–6 and then corroborated the presence of the viroid at a higher level at week 7–8 and 9–10 (Table 3). In one sample, we found an inconsistency. For the analysis conducted at week 5–6, one sample was found negative. The same plant in the subsequent analysis at weeks 7–8 and 9–10 weeks became positive. We speculate either that plant was a false negative or a sampling error occurred during the large-scale sampling in the Set 1 experiment.

**Figure 3:**
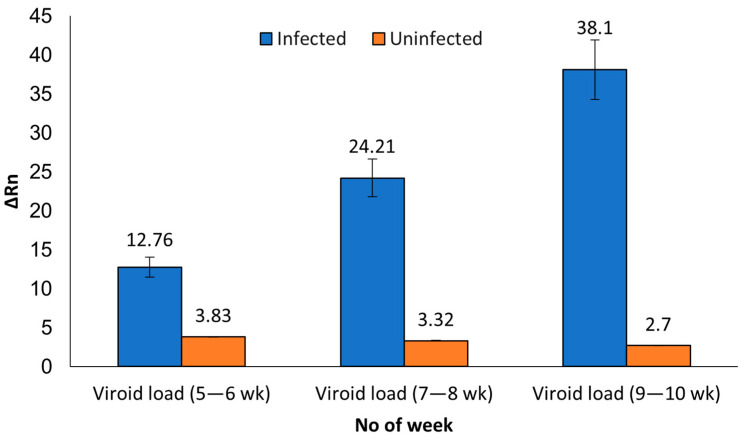
Histogram with the average ΔRn values in the 44 infected (blue bars) and six uninfected (orange bars) plants at different time points starting from 5–6-week-old plants to 7–8-week-old to 9–10-week-old plants.

As we can observe in Figure 3, the HLVd titer in the leaf tissue of the infected plants increased with the age of the plants. To determine the viroid titer in the infected tissue as plants aged, we compared the average ΔRn values for the 5–6-week-old plants with the ΔRn values of the 7–8 week and ΔRn values of the 9–10 week. The ΔRn values increased over time, reaching 1.9× to 3× higher in the older plants. The higher ΔRn values correspond to the higher amounts of the viroid in the older leaf tissue, suggesting the viroid replicated in the leaves. Based on these results, we inferred that the higher load of the viroid makes the leaf an excellent material for HLVd detection. The presence of the viroid in three different tissue types at different titers (ΔRn and Ct values) suggests the viroid load varies with the tissue type and the plant’s age. Our studies further reinforce that once infected, HLVd is persistent in cannabis with the potential to be detected in leaves, petioles and roots using both techniques.

## 4. Discussion

Hop latent viroid (HLVd) is the biggest threat to cannabis growers. The viroid can spread rapidly via mechanical transmission or by unsterilized farm tools, leading to stunted growth, reduced yields, leaf malformation, terpene damage, diminished flower quality, and susceptibility to other pathogens [11,32,33]. HLVd-infected plants can be asymptomatic for a long time; its worldwide occurrence raises great concerns for transmission, as do most viroids [11]. Regular inspection and healthy cultivation management practices are required by the growers to mitigate the large-scale dissemination of the diseases. This becomes critical based on the report that >90% of the cannabis cultivated in California is infected with HLVd. One of the biggest challenges facing the cannabis industry is the timing and identification of infection to minimize the spread of the viroid [34,35]. Thus, a reliable, high throughput sensitive method at lower cost is needed for large-scale testing of the plants in a grow facility and to restrict the spread of viroids. Additionally, the screening will, in turn, produce healthy and high-quality plants for growers.

Various techniques have been used to detect viroid in infected plants, including RT-qPCR and LAMP [25,26,27]. The RT-qPCR assay has been reported to detect different viroids in different plant species [36,37,38,39,40]. Although the efficiency of this assay is high for detection of viroids [41], the specificity of primers and probes makes it complex and not cost-effective for large-scale detection [42,43]. The earliest reports confirming the detection of HLVd in cannabis were also based on RT-qPCR [9,10]. This study describes the MFDetect^TM^ system that relies on a unique set of primers and one-step RT-LAMP plus-qPCR for detecting HLVd in infected plants. A set of oligonucleotides was designed using the program (Primer Explorer 3.0) to detect HLVd successfully. The optimized primer design and concentration in our reaction significantly improved the specificity and expanded the capacity to detect and inspect plant material. Most of the commercial techniques either rely on RT-qPCR, RT-LAMP or other rapid RNA amplification techniques limited by throughput and are expensive compared to the MFDetect^TM^ system.

Previous reports have shown that the addition of intercalating dyes improves the interpretation of the LAMP data and allows real-time monitoring of the amplification reaction. However, the nonspecific binding of these dyes to end products could also increase the risk of false positive calls [44]. Thus, we tested several different dyes in our studies in combination with a new RNA extraction buffer to improve specificity and lower the presence of contaminants to improve the detection. In addition, RNA dilutions up to 40-fold were found to reduce the inhibitory compounds in the RNA extracts. The combination of single-step RT-LAMP and calorimetric detection has been widely used for virus detection in plants and for COVID-19 diagnosis [30,31,45,46,47]. To date, we have analyzed over 150,000 samples using MFDetect^TM^ to detect several other pathogens, including viruses and fungi. We report the most extensive sample used for the detection of multiple pathogens to our knowledge while writing this report.

The sensitivity and accuracy of the MFDetect^TM^ assay was further validated by comparing it with RT-qPCR. Of the fifty plant samples received from our partners, 49 and 50 samples, respectively, were correctly identified as positive or uninfected with RT-LAMP and RT-qPCR methods, respectively. The discrepancies could be because of the extremely low load of the viroid in one of the uninfected plant samples which could be beyond the detection limits of the RT-LAMP assay, or due to a cross-contamination when sampling at week 5–6. The sensitivity of the RT-LAMP for the positive samples was comparable to RT-qPCR. Similar observations comparing the sensitivity of RT-LAMP to RT-PCR have been previously reported [48,49]. In another study, RT-LAMP was shown to be more sensitive than RT-PCR for the detection of six different viroids in Solanaceae [31]. Thus, our findings here are consistent with the previous studies.

Furthermore, a recent report suggested that RT-LAMP was 100 times more sensitive than RT-PCR in detecting the Indian citrus ringspot virus (ICRSV) [50]. The consistency of our results indicates no RNA degradation and no inhibitory compounds in the RNA extraction buffer used for the MFDetect^TM^ assays. Because of the high sensitivity, MFDetect^TM^ may be able to detect infection in the leaves at an early stage of growth before symptoms appear, which is currently a limitation in the industry. Our findings show that an accurate RNA extraction method with a highly sensitive RT-LAMP, as previously suggested [30,50], can highly improve the application of MFDetect^TM^ for viroid detection in cannabis and hemp.

HLVd is mainly transmitted through infected propagules or mechanically through vegetative propagation, infected tools, and grafting [51]. Few studies have reported viroid transmission through pollen and seed [17,52]. After establishing the sensitivity of MFDetect^TM^, we focused our study on the tissue type and age of the plants when determining the best tissue and earliest plant growth stage for HLVd detection. Previous studies have shown that the viroid distribution in plants is not uniform. RT-PCR analyses of the upper, middle, and lower leaves and roots from chrysanthemum showed, in cultivar Piato, that chrysanthemum stunt viroid (CSVd) and chrysanthemum chlorotic mottle Viroid (CChMVd) accumulated almost identically in all tissue types. The authors even found higher titers in bottom leaves than in roots for the cultivar Mari Kazaguruma [53]. In another study, the potato spindle tuber viroid (PSTV) was first detected in the shoot tip, then in leaves next to the shoot tip, followed by inoculated leaf [54]. In citrus, the citrus exocortis viroid (CEVd) and hop stunt viroid (HSVd) titer of each viroid was significant in leaves and roots [55]. In addition, the viroid tomato apical stunt viroid (TASVd) was detected in tomato leaves, roots, and flowers and found to be highly transmitted by seeds [56]. The above findings reinforce that the viroid distribution in plants varies by the type of viroid, tissue, age of plant and cultivar and there is no single tissue type that is best for viroid detection.

In the present study with 600 samples comparing the age and tissue type, we detected the viroid in the leaf samples collected from mother plants from 5–6-week-old cuttings. Our study reports the earliest stage of plant growth for HLVd detection from cannabis. Our future investigation is to enable detection of the viroid in plant cuttings, preferably 1–2-week-old rooted plants. In a subsequent experiment, we observed that leaf, petiole, and root tissue types are equally sensitive to HLVd detection with MFDetect^TM^ and Taqman RT-qPCR. The ease of collecting tissue material and the higher viroid loads in the leaf material make it an ideal material for HLVd detection. It is also reported that HLVd is unevenly distributed in the plants. Based on our findings, we suggest sampling leaves from different plant parts to ensure the infected plants do not go undetected. In fresh cuttings made from infected mother plants for cloning, the viroid likely moves from the leaf and stem to the roots, which emerge first before the new leaves are produced. During root formation, the photosynthate is translocated from the phloem to the root, and therefore the viroid is likely to be found in the roots first (data not proven). The viroid moves through phloem trafficking to lower parts of the plants, from source to sink [57,58]. For mom-cuttings that are 3–4 weeks old plants, roots might be the first tissue to be tested for early detection of HLVd (mainly because leaves have not developed yet). However, uprooting young plants from the substrate is not advisable, and the mechanical harvesting of root segments is not easy. There is the potential to disseminate or cross-contaminate non-infected plants in the process. In addition, there is a significant risk for other opportunistic pathogens, viruses, and viroids to enter through the wounds in the roots. We showed leaves derived from 5-week-old plants (approximately a week post rooting) are suitable for HLVd detection. Our study demonstrates HLVd can be detected in leaf and root material using our sampling protocol with MFDetectTM or by TaqMan RT-qPCR.

This study has several limitations. While in leaf tissue it was easy to extract RNA in the extraction buffer with a tissue-lyser, root and petiole tissues were challenging. The root needed an extra cleaning step while the petiole was difficult to grind. There is room to further improve the protocol for RNA extraction from those demanding tissues. Due to the large sample set, we conducted the MFDetect^TM^ and RT-qPCR assays singly per sample. In the real-world scenario, our commercial lab runs a few thousand tests daily, making it too onerous to completely duplicate every single sample. The study was aimed at evaluating the diagnostic accuracy of MFDetect^TM^ and RT-qPCR in high throughput settings.

In conclusion, we developed a new hybrid RT-LAMP/-qPCR detection system, MFDetect^TM^ for robust detection of HLVd that is rapid, precise, sensitive, and cost-effective. The assays were validated in the most extensive public experiment performed so far from one of the largest and more reputable grower facilities in the USA. Compared to RT-qPCR, MFDetect^TM^ was equally sensitive at detecting HLVd from cannabis plants. To address the growing concerns around tissue type for HLVd detection, we found that all three plant tissues (leaf, petiole, and root) showed the presence of the viroid and gave consistent detection. Because of the ease of sampling, accessibility, and minimal chance of contamination, we recommend that leaf tissues are ideal for HLVd detection. The leaf tissue accumulated higher loads of viroid over time based on the ΔRn values. This further strengthens the justification that leaf is an adequate tissue for early detection. We demonstrate that MFDetect^TM^ is a reliable assay for mitigating the dissemination of the viroid. Follow-up experiments are planned to further narrow the earliest plant growth stage, plant tissue type for detection, and the source of HLVd transmission (for e.g., water, soil or seed) in the grow facilities. When combined with healthy cultivation practices and meristem culture one can produce viroid-free planting material for nurseries and growers.

## Figures and Tables

**Table 1:** Primers used to amplify and detect HLVd in cannabis plants in this study.

Primer Name	Sequence 5′–3′
F3_HLVD_G2	TAAGCTCGGCGCTCAAGA
B3_HLVD_G2	CCCCTCTGGGGAATACACTA
FIP_HLVD_G2	GTTCGCGTCCTGCGTGGAACCCGGGTAGTTTCCAACTCC
BIP_HLVD_G2	GCACGAACTGGCGCTCGATCGTATGGTGGCAAGGGCTC
LF_HLVD_G2	TCCTTCTTCACACCAGCC
LB_HLVD_G2	CTCGCTCGAGTAGGTTTCC

**Table 2:** Comparison of the different plant parts and viroid progression with plant age in the forty-four infected plants selected from initial screening using MFDetect^TM^ and TaqMan RT-qPCR, with their standard error (SE). The 44 plants were selected from an initial screening of 5030 plants.

Tissue Type	Initial Screening (5–6 wk) MFDetect^TM^	Avg ΔRn *	Set 2 (7–8 wk) MFDetect^TM^	Avg ΔRn ± SE	Set 2 (7–8 wk) TaqMan RT-qPCR	Avg CT ± SE	Set 3 (9–10 wk) MFDetect^TM^	Avg ΔRn ± SE	Set 3 (9–10 wk) TaqMan RT-qPCR	Avg CT ± SE
Leaf	44	12.8 ± 0.97	44	24.2 ± 1.2	43	21.9 ± 0.35	44	38.1 ± 2.6	44	19.7 ± 0.5
Petiole	nc	nc	43	27.1 ± 2.0	43	21.3 ± 0.5	43	26.1 ± 2.2	43	21.8 ± 0.3
Root	nc	nc	41	23.8 ± 1.2	44	21.5 ± 0.3	43	19.7 ± 1.6	44	23.0 ± 0.3
Total	44		128		130		130		131	

* In the 1st set of examples only leaf samples were collected and analyzed. Nc = not collected. That represented the first initial screening with 5030 plants.

**Table 3:** Data comparing the accuracy of the MFDetect^TM^ on leaf samples collected from the forty-four infected and six uninfected plants with plant age.

Plant Type	Tissue	Set 1 (5–6 wk) MFDetect^TM^	Set 2 (7–8 wk) MFDetect^TM^	Set 3 (9–10 wk) MFDetect^TM^	MFDetect^TM^ Consistency (Set1, Set2 and Set 3)
Infected	Leaf	44	44	44	99.9%
Uninfected	Leaf	6	5	5	83.3%
Inconsistent	Leaf	0	1	1	-
Total		50	50	50	

## Data Availability

The authors disclose the primer details to benefit the scientific community and the cannabis industry.
